# Association between systolic blood pressure course and outcomes after stroke thrombectomy

**DOI:** 10.1136/bmjno-2021-000183

**Published:** 2021-11-18

**Authors:** Marius Matusevicius, Charith Cooray, Staffan Holmin, Matteo Bottai, Niaz Ahmed

**Affiliations:** 1 Department of Clinical Neuroscience, Karolinska Institutet, Stockholm, Sweden; 2 Department of Research and Education, Karolinska University Hospital, Stockholm, Sweden; 3 Department of Clinical Neurophysiology, Karolinska University Hospital, Stockholm, Sweden; 4 Department of Neuroradiology, Karolinska Universitetssjukhuset, Stockholm, Sweden; 5 Division of Biostatistics, Institute of Environmental Medicine, Karolinska Institutet, Stockholm, Sweden; 6 Department of Neurology, Karolinska University Hospital, Stockholm, Sweden

**Keywords:** stroke, clinical neurology, cerebrovascular

## Abstract

**Background:**

Systolic blood pressure (SBP) after endovascular thrombectomy (EVT) for large artery occlusive stroke is dynamic, requiring adaptable early prediction tools for improving outcomes. We investigated if post-EVT SBP course was associated with outcomes.

**Methods:**

EVT-treated patients who had a stroke at Karolinska University Hospital, Stockholm, Sweden, were included in the study during 12 February 2018–11 February 2020. SBP was recorded during the first 24 hours after EVT. Primary outcome was functional independence defined by a Modified Rankin Scale score of 0–2 at 3 months. Secondary outcomes were death by 3 months, symptomatic intracranial haemorrhage and any intracranial haemorrhage. Patients with favourable outcomes were used as a reference SBP course in mixed linear effects models and compared with SBP courses of patients with unfavourable outcomes using the empirical best linear unbiased predictor, measuring deviations from the reference SBP course using the random effects. We tested model predictive stability for SBP measurements of only 18, 12 or 6 hours after EVT.

**Results:**

374 patients were registered, with mean age 71, median NIHSS score of 15, and 53.2% men. Deviating from a linear SBP course starting at 130 mm Hg and decreasing to 123 mm Hg at 24 hours after EVT was associated with lower chances of functional independence (adjusted OR 0.53, 95% CI 0.29 to 0.88, for reaching either 99 or 147 mm Hg at 24 hours after EVT). All SBP course models for the remaining outcomes did not show statistical significance. Functional independence models showed stable predictive values for all time periods.

**Conclusion:**

Deviating from a linear SBP course was associated with lower chances of 3-month functional independence.

## Introduction

Blood pressure (BP) management after endovascular thrombectomy (EVT) treatment for an acute ischaemic stroke is an important factor for achieving favourable outcomes.^1–4^ Different BP parameters have been investigated for their association with outcomes. Higher mean systolic blood pressure (SBP) values and SBP intervals over 24 hours after EVT treatment were associated with worse outcome.^5 6^ Higher peak SBP values,^7–9^ more BP variability^10 11^ and an increase in SBP during the first 24 hours after EVT were also associated with worse outcomes.^12^ Several of these findings have been confirmed in a meta-analysis.^13^ Yet, these parameters are not individualised and require measurements over a time period in order to know the parameter value, limiting the practical implementation of these parameters. In order to potentially apply an SBP parameter as a predictive tool in clinical practice, it is necessary for it to be both associated with outcomes, known at an early stage, and to some extent individualised. This could be done through careful individual monitoring and targeting of SBP after EVT treatment, as suggested by a previous study.^14^ As SBP after EVT treatment is dynamic, the general SBP course of a patient after EVT treatment could itself be a useful predictor for outcomes. We aimed to investigate if SBP course after EVT treatment in patients who had large artery occlusion stroke could predict favourable outcomes.

## Materials and methods

We collected data from all patients who had larger artery occlusion stroke who underwent EVT treatment at the Karolinska University hospital during 12 February 2018–11 February 2020. Data were collected retrospectively through the electronic patient charts at the hospital. Baseline and demographic characteristics were recorded, in addition to radiological examinations, blood test results, and data on EVT procedural outcomes. Outcomes from 3-month follow-up visits were recorded for all patients who were included during the study period. The data that support the findings of this study are available from the corresponding author on reasonable request.

### Blood pressure

SBP and diastolic BP were recorded at baseline, end of EVT procedure, and during the first 24 hours after EVT treatment. BP was measured according to the local monitoring protocol, which included 21 measurements during the first 24 hours after EVT treatment with measurements every 30 min from 0 hour to 4 hours, measurements every hour from 4 hours to 8 hours, and measurements every 2 hours from 8 hours to 24 hours. Additional eight optional measurements were possible every 2 hours from 9 hours to 23 hours, and these data were collected if they were available. The method for measuring BP was through non-invasive cuffs.

The local guidelines at Karolinska University Hospital recommend using SBP thresholds that are set by the physician performing the EVT procedure. Although individually chosen SBP thresholds were allowed, they largely follow current guidelines,^15^ with a commonly used SBP interval being 100–160 mm Hg for the first 24 hours after thrombectomy.

### Outcomes

The primary outcome was functional independence at 3-month follow-up, defined as a Modified Rankin Scale score of 0–2. Secondary outcomes were death by 3-month follow-up and symptomatic intracerebral haemorrhage (SICH) and any intracerebral haemorrhage (ICH) during routine follow-up radiological examination performed 18–36 hours after EVT treatment. We defined SICH by the Modified Safe Implementation of Treatment in Stroke (mSITS) criteria,^5^ which defines SICH as a local or remote parenchymal haemorrhage type II or any subarachnoid haemorrhage on follow-up radiological examination at 22–36 hours after EVT treatment, in combination with a neurological deterioration of ≥4 points in the National Institute of Health Stroke Scale (NIHSS) or leading to death within 24 hours. We defined any ICH as any sign of a parenchymal haemorrhage type I or II, or subarachnoid haemorrhage on follow-up radiological examination at 18–36 hours after EVT treatment. When creating the prediction models described further, we used patients with favourable outcomes which were patients with functionally independent at 3 months, patients that were alive at 3 months, patients with no SICH by mSITS and patients with no ICH, for each outcome respectively. Patients with missing data on an outcome were excluded from the analyses of that outcome.

### Statistical analysis

Baseline and demographic characteristics were presented for the entire study population and stratified by primary outcome. For group comparisons, Student’s t-test and Pearson’s χ^2^ tests were used for continuous and categorical variables, respectively. We included patients in our analyses if they had at least 10 SBP measurements during the first 24 hours after EVT treatment.

To investigate if deviating from a specific SBP course was associated with outcomes, we created models of SBP measurements over the first 24 hours after EVT treatment for every individual patient, allowing for a linear, quadratic or cubic shape of the SBP course. The initial step for creating each model was to consider the SBP course of patients with favourable outcomes as a reference SBP course, applying the differently shaped curves to their SBP measurements. This SBP course was then compared with patients with unfavourable outcomes. As the shape of the curve of SBP measurements over 24 hours will not match every patient’s SBP measurement exactly, we could test if deviating from the SBP course could itself be associated with outcomes.

Each predictive model was created in three steps, which are summarised as follows and explained in more details further: (1) estimation of the SBP course for patients with favourable outcome using a linear mixed effects model; (2) calculating the random effects for each patient in the entire study population with empirical best linear unbiased predictors (EBLUPs), which provides a value for how much any patient is deviating from the SBP course of patients with favourable outcome; and (3) prediction of the outcome through logistic regression models using the random effects of each patient as the predictor. These steps were based on the previous work by Sandström *et al*.^16^

#### Step 1 of the prediction model

In the first step, we estimated, for each outcome, a linear mixed effects model for the association of SBP over the first 24 hours after EVT in patients who had favourable outcomes. We included random effects for each coefficient of the model, including the intercept, producing the following linear mixed models:

$$SBP_{i,j}=\left(a_{1}+u_{i,0}\right)+\left(b_{1}+u_{i,1}\right)*time+e_{i,j}$$

$$\begin{array}{ll} SBP_{i,j}= & \left(a_{2}+v_{i,0}\right)+\left(b_{2}+v_{i,1}\right)*time+\\ & \left(c_{2}+v_{i,2}\right)*time\hat{2}+e_{i,j} \end{array}$$

$$\begin{array}{ll} SBP_{ij}= & \left(a_{3}+w_{i,0}\right)+\left(b_{3}+w_{i,1}\right)*time\\ & \left(c_{3}+w_{i,2}\right)*time\hat{2}+\left(d_{3}+w_{i,3}\right)*time\hat{3}+e_{i,j} \end{array}$$

for linear, quadratic and cubic versions, respectively, where time was measured in hours. Subscript *i* was for the *i*th patient, and *j* was for the *j*th SBP measurement, as each patient could have a different number of total SBP measurements. The fixed effects *a*, *b*, *c* and *d* were the SBP course parameters for the fixed effect coefficients for the functions’ level, trend, curvature and twist, respectively, where applicable, and differed for each version. The random effects vectors *u=*(*u*_0_, *u*_1_)′, *v=*(*v*_0_, *v*_1_, *v*_2_)′, and *w=*(*w*_0_, *w*_1_, *w*_2_, *w*_3_)′ for the linear, quadratic and cubic versions, respectively, were patient specific. They represented the individual deviation for each patient from the fixed effects of the linear mixed effects model. The residual term *e_i, j_* was assumed to follow a zero-mean normal distribution with variance equal to the SD of the residuals squared (*σ_e_*^2^).

The random effects vectors for each patient were assumed to follow a multivariate normal distribution with mean equal to the *n*-dimensional vector of zeros and covariance matrix (G matrix), where *n* was the length of the random vector, that is, the number of coefficients, in that version of the predictive model:

$$G=\left[\begin{array}{ccc} \gamma_{1,1} & \cdots & \gamma_{1,n}\\ \vdots & \ddots & \vdots \\ \gamma_{1,n} & \cdots & \gamma_{n,n} \end{array}\right]$$

The diagonal *γ_1,1_* to *γ_n, n_* was constrained to be positive, while the remaining values were left unconstrained. The random vectors and the residual *e_i, j_* were assumed to be independent of each other and the time after the EVT procedure.

#### Step 2 of the prediction model

The second step of the predictive models was to obtain the random effects for both patients with favourable and unfavourable outcome, by using the fixed effects found in step 1 as a reference point. Essentially, this was to numerically quantify the deviation of each patient from the fixed effects in step 1. For each patient, we used the EBLUP to estimate the random effects vectors *u*, *v* and *w*, separately, with

$$\begin{array}{l} randomvector=G\overset{´}{Z_{i}}{\left(Z_{i}G\overset{´}{Z_{i}}+\sigma_{e}^{2}I_{i}\right)}^{-1}\left(q_{i}-Z_{i}\alpha \right) \end{array}$$

where *G* was the G matrix, *σ_e_*^2^ was the variance of the residuals; *α* was the vector of fixed effects for the predictive model version; *I_i_* was the identity matrix for a specific patient; *q_i_* was the vector of SBP measurements for a specific patient; and *Z_i_* was a matrix of (time^0^, …, time*^n^*) with *n* being the number of coefficients in that version of the prediction model. While *G*, *σ_e_*^2^ and *α* were the same for all patients within a prediction model version, *Z_i_* and *I_i_* differed in he number of rows for each patient based on the number of SBP measurements for that patient.

Using the EBLUP to acquire the random effects for the entire population has several advantages.^17^ The EBLUP optimally merges the observed SBP values for each patient to the observed values for the population as a whole in order to predict a patient’s trajectory over time. The EBLUP uses shrinkage of the observed residual (*q_i_*−*Z_i_α*) by a factor of *GZ_i_*′(*Z_i_GZ_i_′+σ_e_^2^I_i_*)^−1^ to predict by how much the patient differs from the population trajectory. When the shrinkage factor is large, the predicted trajectory nears the population trajectory. When the shrinkage factor is small, the predicted trajectory nears the observed values for the patient. There are two quantities on which the shrinkage depends: the number of SBP measurements, and the relative magnitude of the variability between patients and within a patient’s measurements. Therefore, the shrinkage factor varies from patient to patient.

#### Step 3 prediction model

The third step of the prediction models involved using values of the random vectors obtained in step 2 in logistic regression models for the outcomes. For each version of the prediction models, all random effects were used in the logistic regression models at the same time. Variables that were deemed clinically relevant according to a directed acyclic graph approach^18^ were also added into the logistic regression models. These covariates were age, sex, baseline NIHSS score, history of hypertension and recanalisation grade by Modified Treatment in Cerebral Infarction (mTICI) score dichotomised as mTICI 2b-3 vs mTICI 0 to 2a.^19^

The three steps described previously are inherently prone to produce possibly underestimated results in step 3, as the steps do not consider any uncertainty in the results from steps 1 and 2. To address this issue, we performed bootstrapping of all the steps for each prediction model version using 1000 iterations per bootstrap. The 95% CIs produced by bootstrapping were presented with the point estimates of step 3.

These three steps were used to compare the SBP courses of two populations, in our case patients with favourable outcomes and patients with unfavourable outcomes. The patients with favourable outcomes were used as a reference SBP course. The reference SBP courses were the fixed effects calculated in step 1, where the SBP course could be modelled from time 0 hour to 24 hours after EVT treatment following the linear, quadratic or cubic shapes for each model. However, even patients with favourable outcome do not necessarily have values that exactly matched the reference SBP course. Therefore, it was necessary to calculate how far from the SBP course an individual may deviate while still predicting favourable outcome. This is measured through the individual random effects of each patient. However, the fixed and random effects from step 1 are known only for the patients with favourable outcomes, meaning that there are no values for patients with unfavourable outcomes. To solve this, we used the EBLUP. The EBLUP uses the fixed effects, in other words, reference SBP course, of the original population to apply the observed values of the patients in the new population as if they were part of the original population using the G matrix and the variance of the residuals of the original population (which represent the variation of the random effects in the original population). This creates random effects for the new population that are essentially the quantified deviation from the reference SBP course of the original population. Because we now have the deviation from the reference SBP course for both patients with favourable and unfavourable outcomes, we can test to see if the deviation itself can be used to predict favourable outcome in logistic regression models.

To compare the prediction models and their predictive stability, we used the area under the curve (AUC) from the receiver operating characteristic curves. In addition to all the SBP measurements that were collected during 24 hours after EVT treatment, we tested the predictive stability of the reference SBP course in different scenarios by using data which contained SBP measurements for only 18, 12 or 6 hours after EVT treatment. Due to fewer SBP measurements in these time scenarios, we included patients with a minimum of 8, 6 and 3 SBP measurements during the time scenarios of 18, 12 and 6 hours, respectively. For these scenarios, the fixed effects, the G matrices and the variance of the residuals remained the same. Due to the different number of SBP measurements within the shorter time scenarios, the random vectors changed for each patient for each scenario.

All statistical analyses were performed with the software R V.4.0.4 (cran.r-project.org).

### Data availability statement

The data that support the findings of this study are available from the corresponding author on reasonable request.

## Results

**Figure 1:**
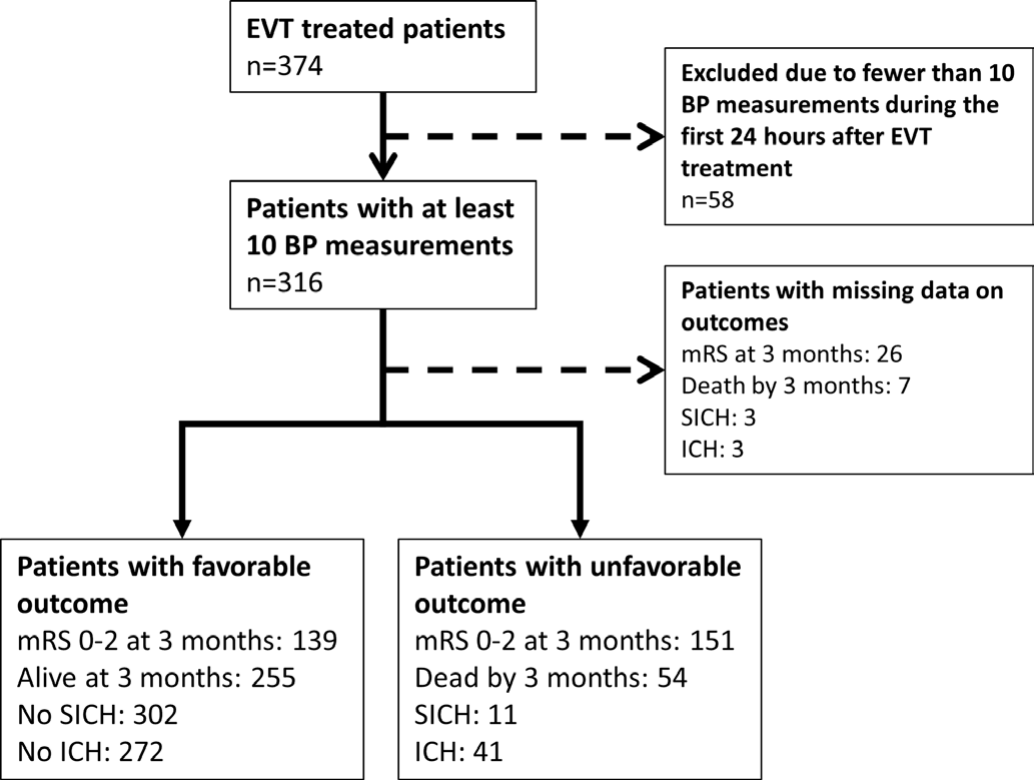
Study flowchart. BP, blood pressure; EVT, endovascular thrombectomy; ICH, intracerebral haemorrhage; mRS, Modified Rankin Scale; SICH, symptomatic intracerebral haemorrhage.

**Figure 2:**
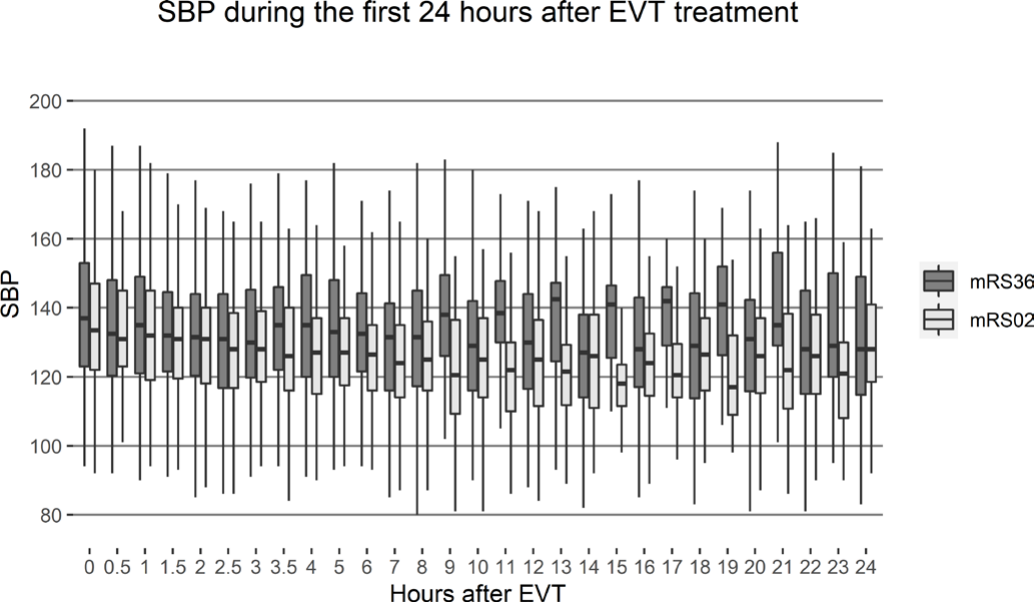
SBP measurements during the first 24 hours after EVT treatment, by 3-month MRS outcome. EVT, endovascular thrombectomy; mRS, Modified Rankin Scale; SBP, systolic blood pressure.

During the study period, 374 patients treated with EVT at our hospital were included in the study (figure 1). The mean age was 71; the median baseline NIHSS score was 15; and 53% of the patients were men (table 1). Patients who achieved functional independence at 3 months were younger (mean 67 years vs 76 years, p<0.001), had lower NIHSS scores (median 13 points vs 18 points, p<0.001), similar proportion of history of hypertension (39% vs 49%, p=0.059) and higher proportion of successful reperfusion (87% vs 70%, p<0.001). The median number of BP measurements was 20 for the 24-hour period, 17 for the 18-hour period, 15 for the 12-hour period and 11 for the 6-hour period after EVT treatment. Compared with patients who were functionally independent at 3 months of follow-up, patients who were dead or dependent had higher values for SBP during the first 24 hours after SBP (figure 2). Among the study participants, 169 (45.2%) achieved functional independence, 292 (78.1) were alive by 3 months of follow-up; 354 (94.7%) had no SICH; and 321 (85.8%) had no ICH. Fifty eight patients (15.5%) had fewer than 10 SBP measurement during the first 24 hours after EVT treatment and were excluded from the analyses.

**Table 1:** Baseline and demographic characteristics and radiological follow-up results

	mRS score 0–2 at 3 months	mRS score 3–6 at 3 months	P value	Missing mRS score at 3 months
n	169	175		30
Age*	66.5 (12.7)	75.8 (12.1)	<0.001	68.6 (10.6)
SBP, baseline*	146.1 (20.8)	150.2 (27.3)	0.139	148.1 (30.9)
Diastolic blood pressure, baseline*	82.8 (14.3)	83.5 (18.5)	0.751	86.9 (20.8)
Glucose, baseline (mmol/L)*	7.6 (5.0)	8.4 (5.1)	0.140	7.6 (2.6)
Cholesterol, baseline (mmol/L)*	4.2 (1.0)	3.9 (1.1)	0.026	4.0 (1.1)
Onset to recanalisation time (min)*	337.6 (239.4)	319.5 (207.1)	0.295	337.2 (180.5)
EVT procedural time (min)*	110.9 (98.8)	129.7 (77.4)	0.084	108.0 (56.7)
NIHSS score, baseline†	13 (7–19)	18 (13–21)	<0.001	14.5 (7.75–19.25)
mRS score, baseline†	0 (0–0)	0 (0–2)	<0.001	0 (0–0)
Sex, male‡	60.9 (103/169)	46.9 (82/175)	0.012	46.7 (14/30)
Platelet inhibitors, baseline‡	16.0 (27/169)	20.6 (36/175)	0.336	23.3 (7/30)
Anticoagulants, baseline‡	18.3 (31/169)	28.0 (49/175)	0.046	6.7 (2/30)
Antihypertensive, baseline‡	55.0 (93/169)	66.3 (116/175)	0.043	46.7 (14/30)
Statin, baseline‡	29.6 (50/169)	32.0 (56/175)	0.713	20.0 (6/30)
Hypertension, baseline‡	38.5 (65/169)	49.1 (86/175)	0.059	40.0 (12/30)
Hyperlipidaemia, baseline‡	6.5 (11/169)	6.3 (11/175)	1.000	10 (3/30)
Diabetes mellitus, baseline‡	8.9 (15/169)	18.3 (32/175)	0.017	16.7 (5/30)
Smoking, baseline‡	24.9 (42/169)	21.7 (38/175)	0.575	16.7 (5/30)
Atrial fibrillation, baseline‡	26.6 (45/169)	33.7 (59/175)	0.189	13.3 (4/30)
Congestive heart failure‡	9.5 (16/169)	14.9 (26/175)	0.173	3.3 (1/30)
Previous TIA‡	4.7 (8/169)	5.7 (10/175)	0.868	3.3 (1/30)
Previous stroke‡	10.1 (17/169)	17.7 (31/175)	0.058	13.3 (4/30)
Aetiology, CE‡	46.2 (78/169)	54.3 (95/175)	0.161	36.7 (11/30)
Aetiology, LAA‡	24.3 (41/169)	26.3 (46/175)	0.758	30.0 (9/30)
Aetiology, other‡	5.9 (10/169)	3.4 (6/175)	0.401	0.0 (0/30)
Circulation anterior/posterior, anterior‡	90.5 (153/169)	88.6 (155/175)	0.676	93.3 (28/30)
ICA‡	7.7 (13/169)	10.9 (19/175)	0.410	3.3 (1/30)
T‡	10.1 (17/169)	15.4 (27/175)	0.184	6.7 (2/30)
M1‡	50.9 (86/169)	50.9 (89/175)	1.000	66.7 (20/30)
M2‡	18.3 (31/169)	10.3 (18/175)	0.047	16.7 (5/30)
Basilaris‡	6.5 (11/169)	6.9 (12/175)	1.000	6.7 (2/30)
ASPECTS†	9 (7, 10)	8 (7, 9)	0.042	8 (7.75, 9)
mCTA†	3 (3, 4)	3 (2, 4)	0.394	3 (3, 4)
IVT treatment‡	53.3 (90/169)	38.9 (68/175)	0.010	50.0 (15/30)
mTICI 2b-3	86.6 (142/164)	69.5 (121/174)	<0.001	26.7 (8/30)
Antihypertensive treatment during 24 hours post-EVT, intravenous‡	17.3 (29/169)	37.1 (65/175)	<0.001	16.7 (5/30)
Antihypertensive treatment during 24 hours post-EVT, oral‡	34.3 (58/169)	20.6 (36/175)	0.006	23.3 (23/30)
Haemorrhagic infarction type I at follow-up of 18–36 hours‡	7.7 (13/168)	10.5 (18/172)	0.493	10.0 (3/30)
Haemorrhagic infarction type II at follow-up of 18–36 hours‡	0.0 (0/168)	2.3 (4/172)	0.137	0.0 (0/30)
Parenchymal haemorrhage type I at follow-up of 18–36 hours‡	3.0 (5/168)	3.5 (6/172)	1.000	6.7 (2/30)
Parenchymal haemorrhage type II at follow-up of 18–36 hours‡	2.4 (4/168)	6.4 (11/172)	0.124	0.0 (0/30)
Remote parenchymal haemorrhage type I follow-up of 18–36 hours‡	1.2 (2/168)	0.6 (1/172)	0.984	0.0 (0/30)
Remote parenchymal haemorrhage type II at follow-up of 18–36 hours‡	0.0 (0/168)	0.0 (0/172)	–	0.0 (0/30)
Subarachnoid haemorrhage at follow-up of 18–36 hours‡	4.2 (7/168)	9.3 (16/172)	0.095	6.7 (2/30)

P value calculated for mRS score of 0–2 vs mRS score of 3–6. For group comparisons of continuous and categorical variables, Student’s t-test and Pearson’s χ^2^ test were used, respectively.

*Mean (SD).

†Median (IQR).

‡Per cent (n cases, n total).

ASPECTS, Alberta Stroke Program Early CT Score; CE, cardiac embolism; EVT, endovascular thrombectomy; ICA, interncal carotid artery; IVT, intravenous thrombolysis; LAA, large artery occlusion; M, middle cerebral artery; mCTA, multiphase CT angiography; mRS, Modified Rankin Scale; mTICI, Modified Treatment in Cerebral Infarction; NIHSS, National Institute of Health Stroke Scale; SBP, systolic blood pressure; T, T-occlusion; TIA, transient ischaemic attack.

**Figure 3:**
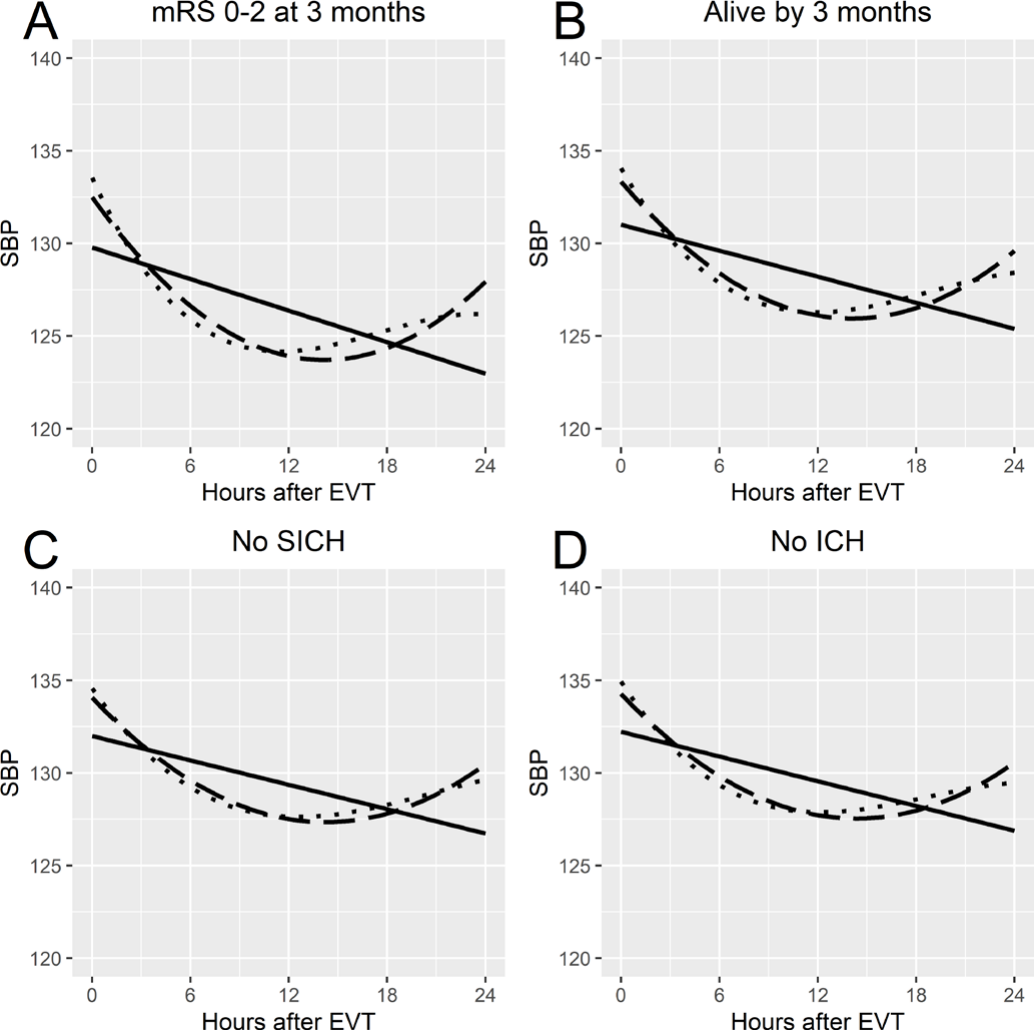
Fixed effects from the mixed effects models for each favourable outcome by prediction model version. (A) mRS score of 0–2 at 3 months. (B) alive by 3 months. (C) No SICH on follow-up radiological examination. (D) No ICH on follow-up radiological examination. Solid line indicates the linear model; dashed line denotes the quadratic model; dotted line indicates the cubic model. EVT, endovascular thrombectomy; ICH, intracerebral haemorrhage; mRS, Modified Rankin Scale; SBP, systolic blood pressure; SICH, symptomatic intracerebral haemorrhage.

The fixed effects of the linear mixed effects models, which are the reference SBP course, showed similar values for the level parameter for each shape of the association but differing values for the remaining SBP course parameters (table 2). For functional independence, the quadratic and cubic shapes of the associations seemed to follow each other well for the first 12 hours (figure 3). This was similar to the fixed effects seen for the remaining outcomes. The G matrices for the linear mixed effects models are shown in online supplemental table 1, and the variance of the residuals is shown in online supplemental table 2.

10.1136/bmjno-2021-000183.supp1 Supplementary data

**Table 2:** Fixed effects for the mixed effects models by prediction model version

Outcome	Models	Level	Trend	Curvature	Twist
mRS score 0–2 at 3 months	Linear model	129.78	−0.28378	–	–
	Quadratic model	132.50	−1.2406	0.04379	–
	Cubic model	133.54	−1.9443	0.12573	−0.00240
Alive at 3 months	Linear model	131.02	−0.23423	–	–
	Quadratic model	133.34	−1.0488	0.03721	–
	Cubic model	134.06	−1.5385	0.09416	−0.00166
No SICH by mSITS	Linear model	131.99	−0.21927	–	–
	Quadratic model	134.06	−0.94195	0.03302	–
	Cubic model	134.57	−1.2899	0.07348	−0.00118
No ICH	Linear model	132.22	−0.22255	–	–
	Quadratic model	134.27	−0.93689	0.03259	–
	Cubic model	134.93	−1.3832	0.08448	−0.00151

ICH, intracerebral haemorrhage; mRS, Modified Rankin Scale; mSITS, Modified Safe Implementation of Treatment in Stroke; SICH, symptomatic intracerebral haemorrhage.

The logistic regression models showed varying results based on the shape of the associations and outcomes (online supplemental table 3). For functional independence at 3 months of follow-up, we observed that deviating from the reference SBP course’s trend parameter for the linear association significantly reduced the chance for functional independence at 3 months of follow-up (adjusted OR 0.53, 95% CI 0.29 to 0.88, when reaching either 99 or 147 mm Hg at 24 hours after EVT). For death by 3 months, SICH by mSITS and any ICH, no model version showed statistically significant results when deviating from the reference SBP course.

AUC values for the logistic regression models for functional independence showed that for SBP measurements during the first 24 hours after EVT, the quadratic and cubic prediction models had the best predictive power (table 3). All versions of the prediction models for functional independence maintained AUC values over 0.8 for all time scenarios. For death by 3 months, all the models showed AUC values of around 0.8 that were stable in all time scenarios but with AUC values lower than the models for functional independence. The models for SICH by mSITS and any ICH showed AUC values below 0.7 in all time scenarios.

**Table 3:** AUC values for the prediction model versions, by hours of SBP measurements testing the models

Outcome	Model	24 hours	18 hours	12 hours	6 hours
mRS score 0–2 at 3 months	Linear model	0.830	0.833	0.835	0.820
	Quadratic model	0.837	0.835	0.836	0.823
	Cubic model	0.837	0.835	0.836	0.823
Alive at 3 months	Linear model	0.799	0.797	0.801	0.795
	Quadratic model	0.801	0.800	0.801	0.795
	Cubic model	0.801	0.800	0.801	0.795
No SICH by mSITS	Linear model	0.636	0.679	0.650	0.662
	Quadratic model	0.633	0.669	0.653	0.667
	Cubic model	0.632	0.669	0.655	0.666
No ICH	Linear model	0.604	0.608	0.600	0.594
	Quadratic model	0.603	0.610	0.605	0.596
	Cubic model	0.628	0.637	0.628	0.612

AUC, area under the curve; ICH, intracerebral haemorrhage; mRS, Modified Rankin Scale; mSITS, Modified Safe Implementation of Treatment in Stroke; SICH, symptomatic intracerebral haemorrhage.

## Discussion

Our study found that deviating from a linearly shaped SBP course based on patients with functional independence during the first 24 hours after EVT treatment for large artery occlusive stroke was significantly associated with a lower chance of functional independence at 3 months. The prediction capacity of this model was stable even when only using SBP measurements for the first 6 hours after EVT.

Understanding how to manage BP after EVT treatment poses several challenges. Only one randomised controlled trial (RCT) has been completed on BP after EVT treatment, where an intervention goal of SBP <130 mm Hg for the first 24 hours after EVT did not reduce intraparenchymal haemorrhage.^20^ The upper limit of SBP in the intervention arm was ambitious, considering the observational data available when the study began enrolment.^1 4 9 21 22^ However, more recent observational data would support SBP thresholds of this level when compared with higher thresholds.^5 23^ Several RCTs are currently enrolling patients for interventions on SBP, with SBP targets ranging from <120 to <140 mm Hg vs controls with SBP <180 mm Hg, where the controls follow current guidelines.^15^ In our reference SBP course models based on patients with favourable outcomes, all of our models suggested SBP values of <140 mm Hg during the first 24 hours after EVT treatment, yet no reference SBP course model suggested SBP values of <120 mm Hg.

Several BP parameters have been shown to be associated with outcomes after EVT treatment in observational studies. Higher peak or maximum values of BP after EVT have been shown to be associated with poor outcomes,^3 7 8^ with one study suggesting that a peak value of SBP >158 mm Hg could be used to discriminate unfavourable outcome.^7^ Additionally, different methods of measuring SBP variation during the first 24 hours after EVT treatment have been investigated, showing an increased risk of unfavourable outcome with increased variation in the form of SBP SD,^11 24^ mean successive variation^10 11 24^ and coefficient of variation.^11 24^ While several BP parameters show associations with outcomes in observational data, most of them share a critical practical drawback, namely, that they are unknown until a period of time has passed. This limits the possibility of using them in clinical practice, as they would not aid the treating physician in making choices during those critical initial 24 hours. In contrast, our prediction models based on a linear SBP course for patients with favourable outcomes showed a stable predictive capacity for functional independence, even when only using SBP measurements for the first 6 hours after EVT, suggesting that the prediction models could be used at an early stage after EVT. Similarly, recent studies have suggested alternative parameters that could potentially be used during the first 24 hours after EVT. One study demonstrated that SBP change from baseline or end of EVT procedure to 0–2, 2–4, 4–12 and 12–24 hours after EVT treatment was associated with worse outcomes, with an even higher risk of worse outcomes for increasing in SBP as opposed to decreasing SBP.^12^ Another study created personalised limits of autoregulation for each patient based on their mean arterial pressure and tissue oxygenation index, which changes based on readings after EVT treatment, showing that going above the upper personalised limits was associated with worse outcomes.^14^ These recent findings suggest that it could be possible to use parameters which are known immediately or shortly after EVT to evaluate a trend or course of BP, with our results potentially adding to this knowledge.

Having a clearly defined and validated SBP threshold after EVT treatment could be an easily applicable approach for SBP management. The focus on SBP thresholds in current RCTs is most likely in part due to the current guidelines, which recommend keeping blood pressure below 180/105 mm Hg after EVT.^15^ The guideline threshold has largely been questioned, with several studies suggesting lower thresholds or intervals in observational data given SBP values over the first 24 hours after EVT.^3 5 23^ While lower thresholds show promising results in observational data, caution should still be used when suggesting them as thresholds for all patients, as these observational studies show that many patients who do not reach BP values under the thresholds still have favourable outcomes. As suggested by Fischer and Mattle,^2^ being more selective as to which patients to treat could provide both better results and more effective treatments. In addition to the SBP parameters themselves, several potential factors have been suggested which affect association of SBP parameters with outcomes after EVT. Reperfusion grade has been shown to affect SBP parameters, with differences in the associations to outcomes based on reperfusion grade.^3 5 24 25^ Additionally, collateral status has also been suggested to affect the association of SBP parameters and outcomes.^24^ In our study, we adjusted our logistic regression models for reperfusion grade. We did not adjust for collateral score, as there was a high number of missing data for this variable (50%). Factors such as infarct site at baseline radiological examination could also be of interest in future prediction models and further on in clinical trials.

This study has some limitations. First, our single-centre study included a modest number of patients that were not based on any power calculation due to a lack of prior data on SBP course. Our more flexible SBP course models may therefore lack statistical power, which may be why some of the more flexible models did not show statistically significant results yet had good predictive capacity. On the other hand, the more flexible models held good predictive capabilities, which could be a sign of overfitting. Second, we did not include information on BP treatments in our statistical models, which could affect both the results and SBP courses we observed. We did not want to add more flexibility in our SBP course models by including the time-specific intervention of BP treatments, but this could potentially hide an effect created by BP-altering treatments. Third, we excluded patients with less than 10 BP measurements during the first 24 hours after EVT in our analyses to improve model stability, but this could potentially add bias when excluding this particular patient subgroup. Finally, we did not add occlusion site, collateral status or Alberta Stroke Program Early CT Score (ASPECTS) in our statistical models. We chose to not include occlusion site due to its even distribution between the modelling populations, and we chose to not include collateral status and ASPECTS due to a high number of missing data. These important factors may affect the models that we presented.

## Conclusion

In conclusion, our single-centre study showed that for patients who had a large artery occlusive stroke who were treated with EVT, deviation from a linear SBP course based on patients with functional independence during the first 24 hours after EVT treatment was associated with lower chances of functional independence at 3 months. We hope to continue to improve and test the predictive models in another patient cohort in order to validate their accuracy.

## Data Availability

Data are available upon reasonable request.
