# Supplementary material for: Association between systolic blood pressure course and outcomes after stroke thrombectomy
Source: BMJ Neurol Open. 2021 Nov 18;3(2):e000183. doi: 10.1136/bmjno-2021-000183 (PMC8603273; doi:10.1136/bmjno-2021-000183)
Supplement: Supplementary data [file bmjno-2021-000183supp001.pdf]

Supplementary material

Supplementary table 1. G-matrices of the prediction models and their versions.						
Outcome	Model	Parameter	Level	Trend	Curvature	Twist
mRS 0-2 at 3 months	Linear model	Level	218.738	-4.55230	-	-
		Trend	-4.55230	0.338246	-	-
	Quadratic model	Level	240.075	-11.2431	0.276963	-
		Trend	-11.2431	2.33825	-0.081741	-
		Curvature	0.276963	-0.081741	0.003294	-
	Cubic model	Level	230.856	-6.77605	-0.140447	0.010503
		Trend	-6.77605	1.04721	0.001639	-0.001577
		Curvature	-0.140447	0.001639	0.001153	-0.000030
		Twist	0.010503	-0.001577	-0.000030	0.000003
Alive at 3 months	Linear model	Level	242.630	-4.52734	-	-
		Trend	-4.52734	0.433319	-	-
	Quadratic model	Level	259.866	-10.7586	0.257936	-
		Trend	-10.7586	2.68435	-0.093166	-
		Curvature	0.257936	-0.093166	0.003809	-
	Cubic model	Level	251.218	-6.20571	-0.173571	0.010673
		Trend	-6.20571	1.26966	-0.003171	-0.001692
		Curvature	-0.173571	-0.003171	0.001685	-0.000033
		Twist	0.010673	-0.001692	-0.000033	0.000004
No SICH by mSITS	Linear model	Level	258.378	-4.94663	-	-
		Trend	-4.94663	0.448198	-	-
	Quadratic model	Level	274.663	-11.2466	0.262574	-
		Trend	-11.2466	2.95507	-0.105089	-
		Curvature	0.262574	-0.105089	0.004358	-
	Cubic model	Level	267.4071	-6.26882	-0.183918	0.010015
		Trend	-6.26882	1.46462	-0.012044	-0.001619
		Curvature	-0.183918	-0.012044	0.00165	-0.000021
		Twist	0.010015	-0.001619	-0.000021	0.000003
No ICH	Linear model	Level	265.941	-5.19221	-	-
		Trend	-5.19221	0.474891	-	-

Matusevicius M, *et al. BMJ Neurol Open* 2022; 3:e000183. doi: 10.1136/bmjno-2021-000183

	Quadratic model	Level	279.567	-10.8473	0.237296	-
		Trend	-10.8473	2.86720	-0.101481	-
		Curvature	0.237296	-0.101481	0.004261	-
	Cubic model	Level	273.290	-6.09015	-0.206795	0.010112
		Trend	-6.09015	1.46621	-0.012655	-0.001575
		Curvature	-0.206795	-0.012655	0.001701	-0.000020
		Twist	0.010112	-0.001575	-0.000020	0.000003
Abbreviations: mRS=Modified Rankin Scale score, SICH by mSITS=Symptomatic intracerebral hemorrhage by modified Safe implementation of treatment in stroke criteria, ICH=Intracerebral hemorrhage.						

Supplementary table 2. Variances of the residuals for each outcome's model version.			
Outcome	Linear model	Quadratic model	Cubic model
mRS 0-2 by 3 months	104.85	93.94	93.04
Alive at 3 months	124.67	113.67	113.03
No SICH by mSITS	130.52	119.07	118.9
No ICH	129.76	118.63	118.32
Abbreviations: mRS=Modified Rankin scale score, SICH by mSITS=Symptomatic intracerebral hemorrhage by modified Safe implementation of treatment in stroke criteria, ICH=Intracerebral hemorrhage.			

Supplementary table 3. Log odds ratios of the logistic regression models for the parameters in the predictions model versions

Outcome	Models	Level	Trend	Curvature	Twist
mRS 0-2 at 3 months	Linear model	-0.0225 (-0.0475 – 0.0026)	-0.6420 (-1.211 – -0.1270)	-	-
	Quadratic model	-0.0212 (-0.0471 – 0.0039)	-0.5260 (-1.126 – 0.0093)	-4.038 (-19.70 – 9.689)	-
	Cubic model	-0.0258 (-0.0909 – 0.0271)	-1.738 (-6.617 – 2.135)	-7.521 (-51.75 – 17.65)	-700.4 (-14007 – 2571)
Alive at 3 months	Linear model	-0.0225 (-0.0532 – 0.0025)	-0.4080 (-1.0279 – 0.1776)	-	-
	Quadratic model	-0.0218 (-0.0523 – 0.0042)	-0.3883 (-1.1148 – 0.2608)	-5.057 (-23.55 – 12.61)	-
	Cubic model	-0.0218 (-0.0681 – 0.0223)	-0.5790 (-5.729 – 3.562)	-5.907 (-58.33 – 40.02)	187.8 (-3872 – 3374)
No SICH by mSITS	Linear model	0.0057 (-0.0630 – 0.0794)	0.3469 (-1.241 – 2.242)	-	-
	Quadratic model	0.0057 (-0.0638 – 0.0854)	0.2734 (-1.379 – 2.477)	4.479 (-41.87 – 68.22)	-
	Cubic model	0.0032 (-0.0857 – 0.1120)	0.5500 (-4.633 – 6.279)	4.081 (-65.86 – 102.7)	-299.4 (-632385 – 1144214)
No ICH	Linear model	0.0074 (-0.0183 – 0.0333)	0.1585 (-0.4903 – 0.8162)	-	-
	Quadratic model	0.0075 (-0.0182 – 0.0335)	0.1428 (-0.5308 – 0.8273)	2.673 (-14.73 – 20.49)	-
	Cubic model	0.0243 (-0.0109 – 0.0610)	-1.809 (-4.638 – 0.9661)	-0.0243 (-17.66 – 17.98)	-1563 (-3870 – 684.9)

Abbreviations: mRS=Modified Rankin Scale score, SICH by mSITS=Symptomatic intracerebral hemorrhage by modified Safe implementation of treatment in stroke criteria, ICH=Intracerebral hemorrhage.
